# Patellar tendon versus artificial grafts in anterior cruciate ligament reconstruction: a systematic review and meta-analysis

**DOI:** 10.1186/s13018-021-02624-x

**Published:** 2021-08-04

**Authors:** DingYuan Fan, Jia Ma, Lei Zhang

**Affiliations:** 1 Beijing University of Chinese Medicine, Beijing, China; 2 Department of Joint Surgery and Sports Medicine, Wangjing Hospital, China Academy of Chinese Medical Sciences, No 6, South Zhonghuan Road, Chaoyang District, Beijing, 100102 People’s Republic of China

**Keywords:** Anterior cruciate ligament, Anterior cruciate ligament reconstruction, Autograft, Artificial, Synthetics, Meta-analysis

## Abstract

**Background:**

The aim of anterior cruciate ligament reconstruction (ACLR) is to restore the function of the knee joint, protect the cartilage, and reduce the occurrence of osteoarthritis. However, due to the structural limitations of the human body, it is not possible to perform ACLR with conventional sutures. To restore normal functioning of the anterior cruciate ligament (ACL), a new ligament must be reconstructed in the position of the previous ACL.

**Objective:**

To compare autografts and synthetic grafts in terms of postoperative knee stability and function

**Search methods:**

The protocol for this study was registered with PROSPERO (CRD42021243451). Two reviewers independently searched the PubMed, Embase, and the Cochrane Library databases from database inception though February 10, 2021. The following search method was used: ((Autograft) OR (Autologous) OR (Autotransplant)) OR Artificial Ligament AND (Anterior Cruciate Ligament Injury [MeSH Terms]) AND (Randomized controlled trial [MeSH Terms]). Methodological quality was assessed by the Cochrane risk of bias tool.

**Selection criteria:**

We only included randomized controlled trials (level I) that compared autograft and synthetic graft interventions in participants with ACL injury. We included trials that evaluated ACLR using at least one outcome (Lachman test, pivot shift test, IKDC grades, or complications).

**Results:**

A total of 748 studies were identified in the initial literature search, and seven studies that examined only bone-patellar tendon-bone (BPTB) grafts compared with artificial grafts met the predetermined inclusion criteria. The results showed that BPTB grafts were associated with significantly better pivot shift test and Lachman test results and better IKDC grades and lower complication rates than synthetic grafts.

**Conclusions:**

This review indicates that for adults, BPTB grafts perform more favorably than synthetic grafts in ACLR in terms of knee stability, function, and complication. More research is needed to compare autologous tendons and allogeneic tendons with artificial ligaments, especially in elderly individuals.

**Level of evidence:**

Level I, systematic review and meta-analysis

## Introduction

In many countries, the incidence of anterior cruciate ligament reconstruction (ACL) injuries has been steadily increasing [1–3]. In addition, the ACL injury rate for women remains 3–6 times greater than that for men and has not changed in over 20 years [4]. Once an ACL injury is diagnosed, the gold-standard surgical procedure for treating ACL injury is performed [5]. In ACLR, the use of different grafts may result in different outcomes, so the surgeon’s selection of grafts is very important. There are three main types of grafts for ACLR: autografts, allografts, and synthetic grafts [5].

Autografts are widely used for ACL because they provide good long-term return to sports results without the risk of graft rejection [6–8]. However, morbidity caused by autograft harvesting and long recovery may affect prognosis [9]. Allografts are another choice for ACLR which is technically easier and not associated with additional donor-site morbidity [10]. However, they are associated with special sterilization techniques, potential infection risk, delayed healing, and higher graft rupture rates [11–15]. In the 1980s, synthetic ligaments were being used in ACL reconstruction to treat ACL injuries [16, 17]. However, these ligaments are associated with high failure rates and reactive synovitis [16–18].

Numerous systematic reviews have compared autografts versus allografts [12, 13, 19, 20]. Joyce et al. [12] showed no difference after ACL reconstruction with nonirradiated BPTB and soft-tissue allografts. Wang et al. [13] reported that the hamstring tendon is superior to allografts in terms of subjective knee evaluations and stability but inferior in terms of hypoesthesia. Prodromos et al. [19] showed that compared with autografts, allografts were associated with significantly less normal stability. Mariscalco et al. [20] showed no significant differences in autografts and allografts. However, only a few systematic reviews and meta-analyses have attempted to determine the superiority of autografts or synthetic grafts [11, 21]. In addition, the studies assessed included nonrandomized, low-quality studies with small sample sizes. A meta-analysis of data from current available studies and quantitative synthesis of their results may provide clarity.

The purpose of this review article was to compare autografts and synthetic grafts in terms of postoperative knee stability and function. The primary outcomes were the pivot shift test, Lachman test, and instrumented laxity. Secondary outcomes were IKDC grades and complications. The authors hypothesized that autografts are superior to synthetic grafts in terms of the pivot shift test, Lachman test, instrumented laxity, IKDC grades, and complications.

## Methods

The Preferred Reporting Items for Systematic Reviews and Meta-Analyses (PRISMA) guidelines [22] were used to extract relevant data from the RCTs included in this meta-analysis. A protocol for the study was registered with PROSPERO (CRD42021243451). The PRISMA checklist was used.

### Search strategy

Two reviewers (DYF, JM) searched the PubMed, Embase, and Cochrane Library databases independently from database inception though February 10th, 2021.The electronic search strategy was as follows: ((Autograft) OR (Autologous) OR (Autotransplant)) OR Artificial Ligament AND (Anterior Cruciate Ligament Injury [MeSH Terms]) AND (Randomized controlled trial [MeSH Terms]). Two reviewers (DYF, JM) screened all the studies identified by title, abstract, and full text using the inclusion criteria. To ensure that no relevant studies were missed, the reference lists of the articles retrieved were also checked. Discrepancies were resolved, by a third reviewer (LZ).

### Eligibility

The inclusion criteria for studies with level I of evidence were as follows: (1) the study only compared autografts vs artificial ligaments and was published before February 10, 2021; (2) the study reported at least one outcome (pivot shift test, Lachman test, IKDC grades, or complication); (3) the article was published in English; (4) the follow-up period was a minimum of 24-months; and (5) if same patients were included in two RCTs, the most recent publication was included. The exclusion criteria were as follows: (1) duplicate reports, (2) reviews and meta-analyses, (3) prospective comparative studies, (4) retrospective comparative studies, (5) case reports and case series, (6) cadaveric reports, (7) cell studies, (8) animal experiment studies, (9) abstract-only publications or full texts that were unavailable, (10) protocols.

### Methodological quality assessment

To assess the methodological quality of the articles included, we used the Cochrane risk of bias tool, which examines 6 domains. Two researchers (DYF, JM) independently performed quality assessments, and a third researcher was consulted if there were any questions.

### Data collection

Two researchers (DYF, JM) extracted the clinical data for this study. Then, the data were reviewed by another researcher (LZ). A spreadsheet that was used to extract the following data comprised of (1) study characteristics, including the year of publication, country in which the study was performed, journal, patient sex, mean patient age (years), follow-up duration (month), cause of the injury, comorbidity, time from injury to surgery (month), and graft type; and (2) pivot shift test, Lachman test results, instrumented laxity, IKDC grades, and complications.

### Statistical analysis

The Cochrane Review Manager statistical software 5.3.0 was used to analyze the extracted data. Dichotomous (pivot shift test, Lachman test, IKDC grades, instrumented laxity, and complications) clinical results are reported as odds ratios (ORs) and 95% confidence interval (CIs). A fixed effects model was used based on our previous assumptions. The *I*^2^ values were calculated and presented in forest plots to quantify the degree of heterogeneity.

The Kappa score was used in this study to assess the level of agreement between reviewers. Scores of 0–0.2, 0.21–0.40, 0.41–0.60, 0.61–0.80, and 0.81–1.00 were considered to indicate slight, fair, moderate, substantial, and almost near perfect agreement, respectively.

#### Subgroup analysis

If data is available, we performed a subgroup analyses, for autografts with different artificial ligaments.

## Results

**Fig. 1:**
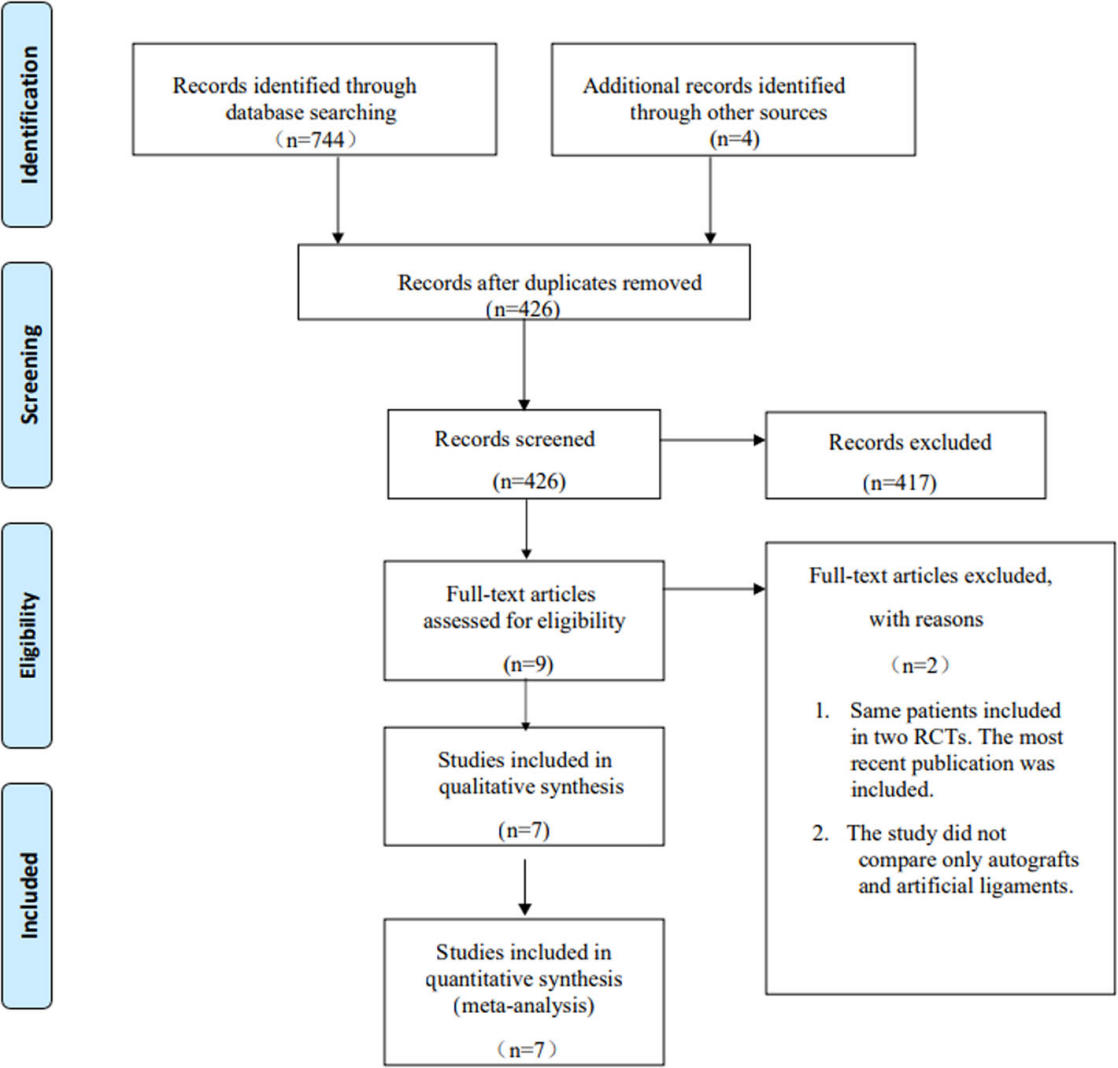
PRISMA flow diagram

From the search of the three online databases, a total of 748 studies were identified, and 426 eligible studies were screened after duplicates were excluded. Two studies were of the same patients with different follow-up times [23, 24], and we included the most recent study. Seven studies [24–30] met the predetermined inclusion criteria. All studies compared bone-patellar tendon-bone (BPTB) grafts with artificial grafts, and no studies evaluating other autografts were identified. The level of agreement between reviewers regarding the inclusion of articles based on the titles was good, and the agreement regarding the inclusion of articles based on the abstracts and full texts was very good. The literature identification and screening results can be found in the PRISMA flow chart (Fig. 1).

### Study characteristics

A total of 504 patients were included in seven studies, and all studies included in this meta-analysis contained level I evidence. The included studies were performed in four countries (Sweden, Norway, Canada, UK) and published in different journals. The follow-up duration ranged from 24 to 300 months (Table 1). The studies reported that soccer play, team handball, and other sporting activities were the main cause of injury. The mean age in the included studies ranged from 23.4 to 31.7 years, and there were no differences in the age or sex distribution among the seven studies (Table 2).

**Table 1:** Overview of included studies

Study	Year	Country	Journal	Follow-up (month)
Engstrom et al. [25]	1993	Sweden	Clinical Orthopaedic and Related Research	28.5
Muren et al. [26]	1995	Sweden	Acta Orthopaedica Scandinavica	48
Grøntvedt and Engebretsen [27]	1995	Norway	Scandinavian Journal of Medicine & Science In Sports	24
Nau et al. [28]	2002	Canada	The Journal of Bone And Joint Surgery	24
Ghalayini et al. [29]	2010	UK	The Knee	60
Peterson et al. [30]	2013	Sweden	Knee Surg Sports Traumatol Arthroscopy	48
Elveos et al. [24]	2018	Norway	The Orthopaedic Journal of Sports Medicine	300

**Table 2:** Overview of included studies

Study	Cause of the injury	Autograft			Synthetic graft		
		Patients	Age	Sex	Patients	Age	Sex
Engstrom et al. [25]	Soccer and other pivoting sports	30	28	M14 F16	30	23.4	M21 F9
Muren et al. [26]	NS	20	25	M13 F7	20	23	M16 F4
Grøntvedt and Engebretsen [27]	Soccer, team handball, and other sporting activities	26	NR	NR	22	NR	NR
Nau et al. [28]	NS	27	30.9	M15 F12	26	31	M21 F5
Ghalayini et al. [29]	NS	26	30.9	M19 F7	24	31.7	M21 F3
Peterson et al. [30]	NS	86	27	35	74	27	49
Elveos et al. [24]	Soccer, team handball, and other sporting activities	48	25	NR	45	27	NR

*NS* not shown

### Outcomes

Five studies used the Lachman test [24, 25, 27, 29, 30]. Five studies reported complications [24, 26–28, 30]. Four studies used the pivot shift test [24–27, 30], and four studies reported IKDC score [25, 28–30] (Table 3).

**Table 3:** Overview of included studies

Study	Comorbidity	Time from injury to surgery (month)	Graft type	Outcome
Engstrom et al. [25]	Medial collateral ligament injury, medial meniscus injury, lateral meniscus injury	32.5	Bone-patellar tendon-bone graft Leeds-Keio graft	Pivot shift test, Lachman test, IKDC;
Muren et al. [26]	Medial collateral ligament suture, medial meniscus surgery, lateral meniscus surgery, extra-articular reconstruction	30	Bone-patellar tendon-bone graft Kennedy ligament augmentation device	Pivot shift, instrumented, complications
Grøntvedt and Engebretsen [27]	Meniscal ruptures, medial collateral ligament injuries	NS	Bone-patellar tendon-bone graft Kennedy ligament augmentation device	Lachman test, instrumented, pivot shift, complications;
Nau et al. [28]	Meniscal tears	57.6	Bone-patellar tendon-bone graft Ligament Advancement Reinforcement System	Instrumented, IKDC, complications
Ghalayini et al. [29]	Meniscal pathology	NS	Bone-patellar tendon-bone graft Leeds-Keio graft	IKDC, Lachman test
Peterson et al. [30]	NS	16 (augmentation) 24 (patellar tendon)	Bone-patellar tendon-bone graft Poly (urethane urea) augmentation device (Artelon)	Lachman test, pivot shift test, IKDC, instrumented, complications
Elveos et al. [24]	NS	40	Bone-patellar tendon-bone graft Kennedy ligament augmentation device	Lachman test, pivot shift test, instrumented, complications

*NS* not shown, *IKDC* International Knee Documentation Committee

### Methodologic quality assessment

**Fig. 2:**
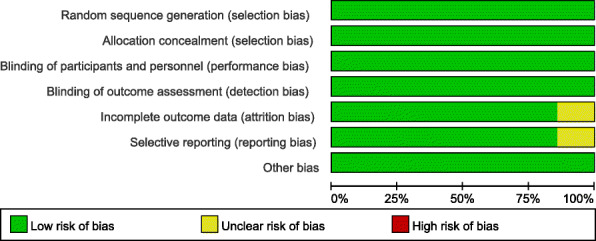
Risk of bias graph

**Fig. 3:**
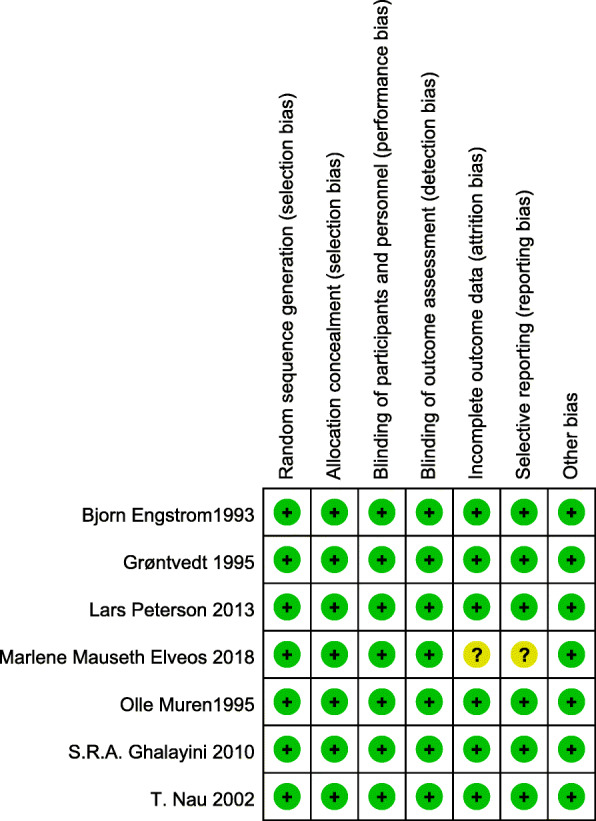
Risk of bias summary

This study used the Cochrane Collaboration risk of bias tool to evaluate the risk of bias of the six randomized studies. The sequence generation and allocation methods were reported for all included studies. All studies included blinding of the outcome assessor, and as a result, a low risk for detection bias was noted. Seven studies had a low risk of bias because all patients were blinded to the intervention. Only two studies reported significant loss of follow-up rates. Four studies had a low risk regarding selection bias and incomplete outcome data reporting (Figs. 2 and 3). Very good study agreement was reported Kappa score =0.88.

### Pivot shift test

**Fig. 4:**
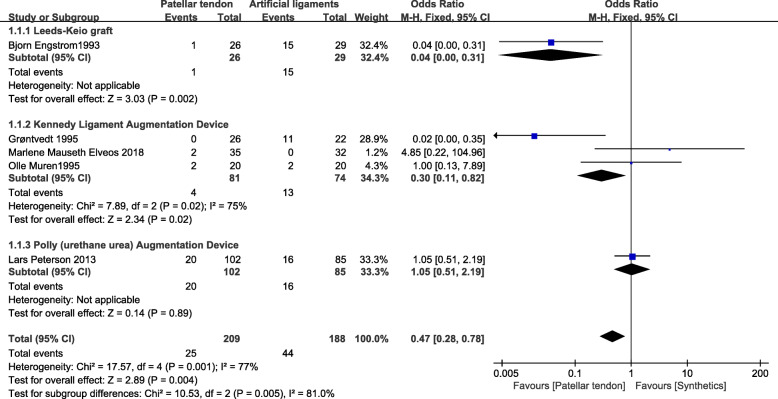
Pivot shift test forest plot

Five studies reported pivot shift test results [24–27, 30]. A total of 397 patients were included in the two groups (Fig. 4). In the Leeds-Keio graft subgroup, poor data showed that the BPTB group had lower pivot shift positive rate than the Leeds-Keio graft (OR=0.04; 95% CI 0.00, 0.31). However, in the poly (urethane urea) augmentation device (Artelon) subgroup, the BPTB graft group showed no significant difference from the synthetic group (OR=1.05; 95% CI 0.51, 2.19). In the Kennedy ligament augmentation device subgroup, compared with BPTB grafts, artificial grafts had poor results (OR=0.30; 95% CI 0.11, 0.82; *p*=0.02; *I*^2^=75%). Similarly, BPTB grafts had better result than synthetic grafts (OR=0.47; 95% CI 0.28, 0.78; *p*=0.001, *I*^2^=77%). The test for subgroup differences showed high heterogeneity (*I*^2^=0.81).

### Lachman test

**Fig. 5:**
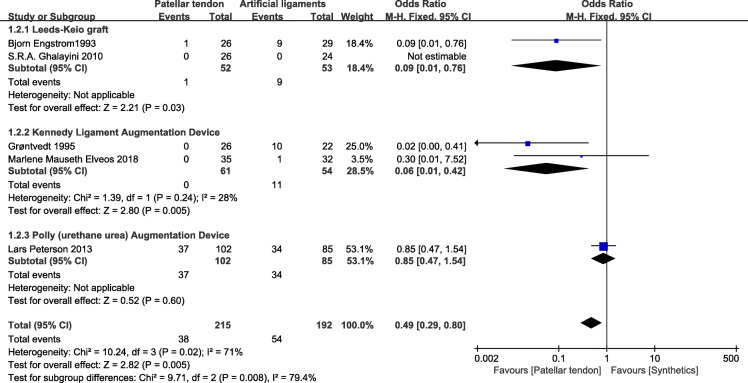
Lachman test forest plot

Five studies reported cramping pain [24, 25, 27, 29, 30]. There were 215 patients who used patellar tendons and 192 patients who used synthetics (Fig. 5). In the Leeds-Keio graft subgroup, the poor data showed less Lachman test positivity in the BPTB group (OR=0.09; 95% CI 0.01, 0.76). Similarly, in the Kennedy ligament augmentation device subgroup, compared with BPTB grafts, artificial grafts had worse results (OR=0.06; 95% CI 0.01, 0.42; *p*=0.24; *I*^2^=28%). Conversely, the poly (urethane urea) augmentation device (Artelon) showed no significant difference from the BPTB group (OR=0.85; 95% CI 0.47, 1.54). Collectively, the 215 patients in BPTB group showed lower positive Lachman test positive rate compared with the 192 patients in the synthetic graft group (OR=0.49; 95% CI 0.29, 0.80; *p*=0.02; *I*^2^=71). The test for subgroup differences indicated the presence of heterogeneity (79.4%).

### Instrumented laxity

**Fig. 6:**
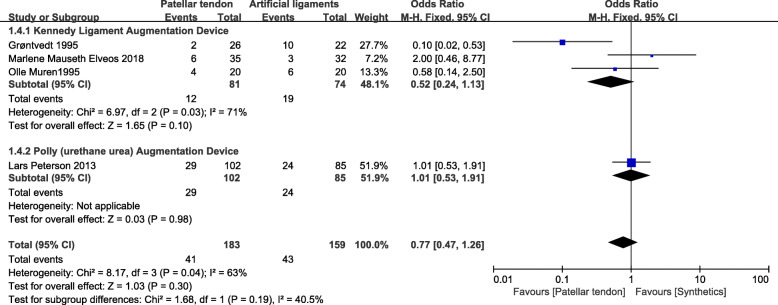
Instrumented forest plot

Four studies [24, 25, 27, 30] that included 342 patients (183 patients treated with patellar tendons and 159 patients treated with synthetics) reported instrumented laxity (>3mm). In the Kennedy ligament augmentation device subgroup, the data showed no significant difference between the BPTB group and the synthetics group (OR=0.52, 95% CI 0.24, 1.13; *I*^2^=71%). Similarly, the poly (urethane urea) augmentation device (Artelon) group showed no significant difference from the BPTB group (OR=1.01; 95% CI 0.53, 1.91). Collectively, the 183 patients in BPTB group showed no significant difference from the 159 patients in the synthetic graft group (OR=0.77; 95% CI 0.47, 1.26; *p*=0.02; *I*^2^=63%). The test for subgroup differences indicated the presence of heterogeneity (40.5%) (Fig. 6).

### IKDC grades

**Fig. 7:**
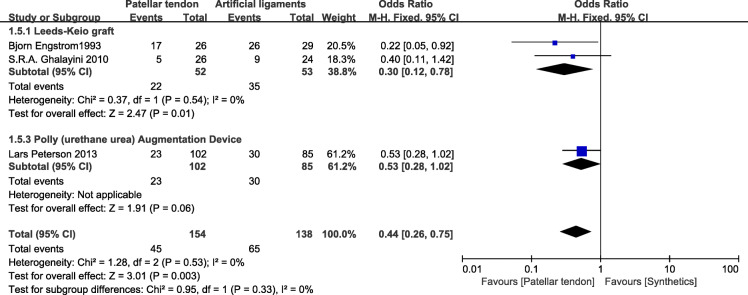
IKDC grades forest plot

Three studies [25, 29, 30] that included 292 patients (154 patients treated with patellar tendons and 138 patients treated with synthetics) reported IKDC grades. One study was excluded because of different data types [28]. In the Leeds-Keio graft subgroup, the data showed better IKDC grades in the BPTB group than in the synthetic graft group (OR=0.30; 95% CI 0.12, 0.78). In the poly (urethane urea) augmentation device (Artelon) subgroup, the pooled data for artificial grafts showed no significant difference from those for BPTB grafts (OR=0.53; 95% CI 0.28, 1.02). Conversely, 176 patients in the BPTB group showed better IKDC grades than 164 patients in the synthetic graft group (OR=0.44; 95% CI 0.26, 0.75; *p*=0.53; *I*^2^=0). No heterogeneity for subgroup differences was found (Fig. 7).

### Complications

**Fig. 8:**
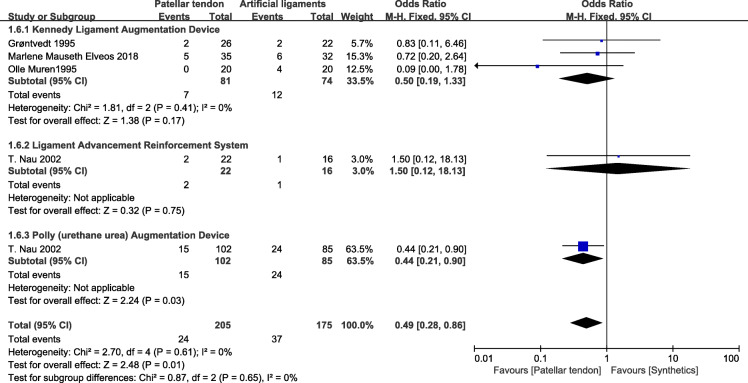
Complications forest plot

Five studies [24, 25, 27, 28, 30] that included 380 patients (205 patients treated with patellar tendons and 1175 patients treated with synthetics) reported complications. In the Leeds-Keio graft subgroup, three studies showed no significant difference between the two groups (OR=0.50; 95% CI 0.19, 1.33). Similarly, in the Ligament Advancement Reinforcement System (LARS) subgroup, the synthetic graft group showed no significant difference with BPTB group (OR=1.50; 95% CI 0.12, 18.13). In the poly (urethane urea) augmentation device (Artelon) subgroup, compared with BPTB grafts, artificial grafts showed worse results (OR=0.49; 95% CI 0.28, 0.86). Collectively, 205 patients in the BPTB group showed superior results compared with 175 patients in the synthetic graft group (OR=0.49; 95% CI 0.28, 0.86; *p*=0.61; *I*^2^=0%). No heterogeneity was found according to the test for subgroup differences (Fig. 8).

## Discussion

In this meta-analysis, the most important finding was that BPTB grafts were associated with better pivot shift, Lachman test results, and IKDC grades and fewer complications than synthetics.

In this study, we found that in the pivot test (OR=0.47; 95% CI 0.28, 0.78) and Lachman test (OR=0.49; 95% CI 0.29, 0.80), BPTB grafts were associated with better results than synthetics. The Kennedy ligament augmentation device (Kennedy LAD) and Leeds-Keio grafts were also associated with worse in pivot test and Lachman test results, which is similar to the findings of a previous study [11]. Jia et al. [11] showed that Kennedy LAD and Leeds-Keio grafts had worse results in terms of instrumented laxity, but our study found there was no difference between the two groups (OR=0.77; 95% CI 0.47, 1.26). This result may be due to our inclusion of the study with the longer follow-up of the same patients. In contrast, Sun et al. found lower instrumented laxity with a Ligament Augmentation and Reconstruction System (LARS) [21]. IKDC grades, a widely used tool for assessing knee function and pathology, were better for BPTB grafts than old-generation synthetic grafts (OR=0.30; 95% CI 0.12, 0.78), and this result was supported by Jia et al. [11]. After surgery, complications are an important problem that needs to be addressed. In this study, the Leeds-Keio graft (OR=0.50; 95% CI 019, 1.33) and Kennedy LAD subgroups (OR=1.50, 95% CI 0.12, 18.13) showed no significant differences in complications between the two groups. However, the overall results show that autogenous tendons remain the preferred option (OR=0.49; 95% CI 0.28, 0.86) because of the poly augmentation device. In the Sun et al. study, autografts had a higher rate of complications than LARSs, which may indicate an improvement in new artificial ligaments compared to older generation ligaments [21].

Artificial grafts became popular for ACL reconstruction in 1980s [16, 17]. They provide greater strength and stability and decreases donor site morbidity and the risk of disease transmission [5, 17]. Second-generation artificial ligaments include longitudinal and transverse fibers to promote fibroblastic ingrowth as scaffoldings but still cause wear and debris [31]. A LARS is a nonabsorbable polyethylene terephthalate graft [32]. It is a third-generation synthetic ligament and attempts to provide a meshwork for repair and avoid the complications of reactive synovitis [18]. As one of the commonly used artificial ligaments, its clinical efficacy has been affirmed. A multicenter study reported by Gao et al. found that LARSs used in the acute and chronic phases had good outcomes with a low rate of complications [33]. Bugelli et al. found that a total of 31.25% of included patients were able to resume their lifestyle from before the injury, and the subjective evaluation showed good/excellent results [34]. A 10-year longitudinal study reported that primary ACLR using synthetics showed satisfactory outcomes [35]. In 2018, Parchi et al. found that for elderly patients, using a LARS ligament can be a safe and suitable option and enable a rapid postoperative recovery [36]. In 2019, ACLR was reported to be associated with good knee function scores, a high rate of return to sport, and low rates of re-rupture [37]. Tsai et al. reported that knee stability improved immediately after ACLR with a LARS [38]. Su et al. reported no statistically significant differences among allografts, 4-strand hamstring tendon autografts, and LARSs in terms of the clinical outcomes after ACLR [39].

## Authors’ conclusions

### Implications for practice

This review indicates that for adults, BPTB grafts are more favorable than synthetic grafts in ALCR in terms of knee stability, function, and complication rates. The high-quality evidence of these results is similar to that of those from the previous version of this review, as no new randomized trials have been conducted.

However, the conclusions of this review do not apply to older populations because no elderly people were included in these studies.

#### For people with ACLR

For adults, BPTB grafts are associated with better knee function, stability degree, and complication than synthetic graft.

#### For clinicians

BPTB is still the “gold standard” for ACLR and provides better knee stability, function, and complication rates than synthetic grafts in adults.

#### For policy makers

BPTB is an effective autograft, compared with synthetics, for adults in ACLR.

### Implications for research

#### General

We found major limitations in the current evidence base. All randomized controlled studies that we included compared only BPTB grafts with artificial ligaments, and only 1 to 3 studies were included for each type of artificial graft. It was difficult to thoroughly compare BPTB grafts with specific types of artificial ligaments. More importantly, the mean age of all the patients included in the literature was less than 32 years, which made it impossible to evaluate the efficacy of BPTB grafts and artificial ligaments in elderly individuals. In addition, comparison of the efficacy of other autogenous tendons or allogeneic tendons with artificial ligaments was not possible with the included studies.

We suggest the following investigation guidelines to help further discussions in this area.

Patients who are elderly and undergoing ACLR and reconstruction of other ligaments of the knee joints need to be considered.

Interventions need to consider other autologous tendons

- Comparisons need to consider the latest generation of ligaments in the clinic.
- Outcomes should include subjective function scores, quality of life, re-rupture, and return to activity or sport.
- The final follow-up time should be 2 years or more
- Reporting of randomized trials should follow the Consolidated Standards of Reporting Trials (CONSORT) guidelines.

## Data Availability

The present study was a review of previously published literature.

## Abbreviations

ACLR: Anterior cruciate ligament reconstruction
BPTB: Bone-patellar tendon-bone
ACL: Anterior cruciate ligament
RCTs: Randomized controlled trials
PRISMA: Preferred Reporting Items for Systematic Reviews and Meta-Analyses
CI: Confidence interval
MD: Mean difference
OR: Odds ratios
KOOS: Knee injury Osteoarthritis Outcome Score
LARS: Ligament Advancement Reinforcement System
HT: Hamming tendon
Kennedy LAD: Kennedy ligament augmentation device
